# Comparison of three-parameter kinetic model analysis to standard Patlak’s analysis in ^18^F-FDG PET imaging of lung cancer patients

**DOI:** 10.1186/s13550-018-0369-5

**Published:** 2018-03-27

**Authors:** E. Laffon, M. L. Calcagni, G. Galli, A. Giordano, A. Capotosti, R. Marthan, L. Indovina

**Affiliations:** 1 Departments of Nuclear Medicine & Lung Function Testing, CHU de Bordeaux, Bordeaux, France; 2 Centre de Recherche Cardio-Thoracique, INSERM U-1045, Univ. Bordeaux, Bordeaux, France; 3 Physics Unit, Fondazione Policlinico Universitario “A. Gemelli”, Roma, Italy; 4 Institute of Nuclear Medicine, Università Cattolica del Sacro Cuore, Roma, Italy; 5 Institute of Physics, Università Cattolica del Sacro Cuore, Roma, Italy; 6 Service de Médecine Nucléaire, Hôpital du Haut-Lévêque, Avenue de Magellan, 33604 Pessac, France

**Keywords:** ^18^F-FDG PET, Patlak’s analysis, Reversible trapping, Uptake/release rate constant, lung cancer

## Abstract

**Background:**

Patlak’s graphical analysis can provide tracer net influx constant (Ki) with limitation of assuming irreversible tracer trapping, that is, release rate constant (k_b_) set to zero. We compared linear Patlak’s analysis to non-linear three-compartment three-parameter kinetic model analysis (3P-KMA) providing Ki, k_b_, and fraction of free ^18^F-FDG in blood and interstitial volume (V_b_).

**Methods:**

Dynamic PET data of 21 lung cancer patients were retrospectively analyzed, yielding for each patient an ^18^F-FDG input function (IF) and a tissue time-activity curve. The former was fitted with a three-exponentially decreasing function, and the latter was fitted with an analytical formula involving the fitted IF data (11 data points, ranging 7.5–57.5 min post-injection). Bland-Altman analysis was used for Ki comparison between Patlak’s analysis and 3P-KMA. Additionally, a three-compartment five-parameter KMA (5P-KMA) was implemented for comparison with Patlak’s analysis and 3P-KMA.

**Results:**

We found that 3P-KMA Ki was significantly greater than Patlak’s Ki over the whole patient series, + 6.0% on average, with limits of agreement of ± 17.1% (95% confidence). Excluding 8 out of 21 patients with k_b_ > 0 deleted this difference. A strong correlation was found between Ki ratio (=3P-KMA/Patlak) and k_b_ (*R* = 0.801; *P* < 0.001). No significant difference in Ki was found between 3P-KMA versus 5P-KMA, and between 5P-KMA versus Patlak’s analysis, with limits of agreement of ± 23.0 and ± 31.7% (95% confidence), respectively.

**Conclusions:**

Comparison between 3P-KMA and Patlak’s analysis significantly showed that the latter underestimates Ki because it arbitrarily set k_b_ to zero: the greater the k_b_ value, the greater the Ki underestimation. This underestimation was not revealed when comparing 5P-KMA and Patlak’s analysis. We suggest that further studies are warranted to investigate the 3P-KMA efficiency in various tissues showing greater ^18^F-FDG trapping reversibility than lung cancer lesions.

## Background

Positron emission tomography using [^18^F]fluorodeoxyglucose (^18^F-FDG PET) imaging in oncology patients allows physicians to quantify the increased glycolysis of cancer cells [1]. In clinical routine, a tracer uptake index is easily available and thus widely used, namely, the standardized uptake value (SUV) [2, 3]. However, many factors can influence the SUV outcome such as the uptake time, as reported for example in lung tumors [4]. This is the reason why, besides the SUV index, different quantitative parameters that may be obtained from kinetic model analyses (KMAs) have been implemented in a number of studies investigating various tissues [5–13]. These kinetic parameters more accurately describe the tracer trapping and may be useful to better characterize different tumor types or assess treatment response [14]. The KMAs both require a dynamic acquisition over the tissue of interest to obtain its time-activity-curve (TAC) and a serial blood sampling to estimate the so-called input function (IF, i.e., ^18^F-FDG blood TAC). Among these KMAs, Patlak’s analysis is usually considered as a gold standard that provides the ^18^F-FDG net influx constant (i.e., the uptake rate constant, Ki) from a linear fitting of graphical data [7]. However, it assumes an irreversible tracer trapping, a well-identified drawback since numerous studies have shown trapping reversibility in various tissues, either under physiological or pathological conditions [6, 9–11, 15].

Assuming that there may be a slow loss of the trapped tracer to the blood, i.e., a reversible trapping, Patlak and Blasberg derived a generalized non-linear equation including a release rate constant (k_b_) in an exponential term [8]. This non-linear equation may be addressed by using an analytical approach, leading to a three-compartment three-parameter KMA (3P-KMA). 3P-KMA has been applied to healthy human lung and liver, allowing assessment of both Ki (in mL min^−1^ mL^−1^), k_b_ (in min^−1^), and fraction of free ^18^F-FDG in blood and interstitial volume (V_b_; no unit; < 1; also called total blood volume distribution) [12, 15]. It relies on an analytical solution  of the non-linear Patlak’s equation that requires to use an IF as a sum of exponentially decreasing functions and up to three functions may usually describe the ^18^F-FDG IF [16, 17]. Then, it leads to a non-linear formula that is used to fit the experimental tissue TAC by simply adjusting the three above-mentioned kinetic parameters, i.e., without any tissue TAC data transform.

To the best of our knowledge, comparison between non-linear fitting by 3P-KMA and linear fitting by standard Patlak’s analysis that assumes an irreversible tracer trapping has not been reported so far, whatever the tissue either under physiological or pathological conditions. Therefore, the primary aim of this study was to make this comparison in a series of lung cancer patients that was previously acquired [13]. Additionally, Ki, k_b_, and V_b_ outcomes obtained from 3P-KMA were compared to those obtained from a three-compartment five-parameter KMA (5P-KMA) that is usually considered as a reference model when tracer trapping is reversible. Actually, 5P-KMA provides four kinetic (micro)parameters (and V_b_) from which Ki and k_b_ may be computed, whereas the non-linear fitting of the 3P-KMA provides Ki and k_b_ without any additional computing.

## Methods

### Patients

Dynamic data of 21 patients (8 females, 13 males, 71 years old on average, range 40–86) with non-small cell lung cancer obtained from a previous prospective study were retrospectively analyzed [13]. All patients who were enrolled in the prospective study provided written informed consent before participating in it, and the further retrospective study received the approval of the ethics committees of our teaching hospitals. The patients’ mean weight and height were 68 kg (range, 50–85) and 169 cm (range, 150–180), respectively. After 6 h of fasting before the tracer injection, the preinjection average plasma glucose concentration was 1.08 g L^−1^ (range, 0.87–1.28). The lesion mean size was 31.8 mm (range, 14.7–52.2).

### PET imaging and data processing

PET imaging procedure has been previously described in details [13]. Briefly, a low dose CT scan was performed (75 mA, 120 kV, pitch 0.938, rotation time 0.5 s) for attenuation correction of PET emission data and for morphologic information. Then, after an intravenous bolus injection of ^18^F-FDG (mean 237 MBq; range, 134–507) in a cannula previously inserted in the vein of the arm, a 3D thorax dynamic list-mode acquisition protocol was started lasting 60 min (Gemini GXL, Philips Medical System, Cleveland, USA; no respiratory gating). Images were reconstructed using the iterative method RAMLA LOR-3D, with a 144 × 144 matrix and pixel size of 4 × 4 × 4 mm^3^. In particular, this dynamic acquisition provided 11 frames of 5 min each, leading to 11 data points of the experimental ^18^F-FDG IF and of the experimental cancer tissue TAC, ranging 7.5–57.5 min post-injection. For determining the experimental ^18^F-FDG IF, in each patient, a volume of interest (VOI) was drawn over the descending thoracic aorta in each frame of the dynamic acquisition yielding an intermediate ^18^F-FDG blood TAC (i.e., intermediate IF). Then, the final IF was obtained through a calibration of the intermediate IF with the ^18^F-FDG plasma value measured in a venous blood sampling performed at 45 min post-injection, that is, when an equilibrium is reached between ^18^F-FDG concentration in arterial and vein blood [3, 16]. VOIs for IF and tissue TAC were semi-automatically placed over three consecutive slices to include the five hottest voxels within the VOI.

### Implementing Patlak’s analysis, 3P-KMA, and 5P-KMA

Assuming that there may be a slow loss of the trapped tracer to the blood and when the analysis remains limited to data collected for the period t > t* after injection, that is, when the reversible compartments are in effective steady state with the blood plasma, Patlak and Blasberg derived a non-linear equation including a release rate constant (k_b_) [8]:

1 $$ {\mathrm{A}}_{\mathrm{T}}\left(\mathrm{t}\right)/{\mathrm{A}}_{\mathrm{p}}\left(\mathrm{t}\right)=\left[\mathrm{Ki}{\int}_{\mathrm{o}}^{\mathrm{t}}{\mathrm{A}}_{\mathrm{p}}\left(\uptau \right){\mathrm{e}}^{\hbox{-} {\mathrm{K}}_{\mathrm{b}}\left(\mathrm{t}\hbox{-} \uptau \right)}\mathrm{d}\uptau \right]/\left[{\mathrm{A}}_{\mathrm{p}}\left(\mathrm{t}\right)\right]+{\mathrm{V}}_{\mathrm{b}} $$

A_T_(t) (in kBq mL^−1^) is defined as the total tracer activity at time t per tissue volume unit that includes both trapped tracer and free tracer in the blood and interstitial volumes. A_p_(t) (in kBq mL^−1^) is the blood activity at time t per blood volume unit, that is, the ^18^F-FDG IF.

In each patient, Patlak’s graphical analysis was implemented from Eq. 1, setting k_b_ = 0. Eleven cancer tissue TAC data points and the corresponding 11 data points of the experimental ^18^F-FDG IF, ranging 7.5–57.5 min post-injection, were used. The lower limit of 7.5 min for this range was chosen in order to limit the analysis to data collected for the period t > t* after injection, as required by Eq. 1 validity [7, 8]. Ki was determined as the slope of the linear fitting of the Patlak’s plot showing A_T_(t)/A_p_(t) versus the ratio of time integral of the right hand side of Eq. 1 to A_p_(t), i.e., the so-called stretched time.

In each patient, 3P-KMA was implemented by first fitting the 11 data points of the experimental ^18^F-FDG IF with a three exponentially decreasing function derived from Hunter’s results, after data were uncorrected for the ^18^F physical decay. Hunter’s results were used, and not Vriens’ ones as in previous studies, because the former were established with blood sampling performed at 55 min post-injection, in comparison with 25 min for the latter [12, 15–17]:

2 $$ {\mathrm{A}}_{\mathrm{p}}\left(\mathrm{t}\right)={\mathrm{A}}_0\times \left[8.20\times \exp \left(\hbox{-} 9.3363\times \mathrm{t}\right)+1.17\times \exp \left(\hbox{-} {\upalpha}_2\times \mathrm{t}\right)+\exp \left(\hbox{-} {\upalpha}_3\times \mathrm{t}\right)\right] $$

In Eq. 2, the amplitude ratios of the three exponential functions and the time constant of the first exponential function (uncorrected for physical decay) were available from Hunter’s results [16]. In each patient, *A*_0_ (leading to virtual initial IF amplitude), α_2_, and α_3_ (time constants of the second and third exponential functions, uncorrected for physical decay) were obtained by fitting the experimental ^18^F-FDG IF data points (XLSTAT Microsoft; Levenberg-Marquardt algorithm). Then, in each patient, a formula was established by analytically solving integral of Eq. 1 and by using the fitted three-exponential IF of Eq. 2 involving k_b_ [12, 15]:

3 $$ {\displaystyle \begin{array}{ll}{\mathrm{A}}_{\mathrm{T}}\left(\mathrm{t}\right)=\mathrm{Ki}\times {\mathrm{A}}_0& \times \Big\{8.20\times \left[\exp \left(\hbox{-} 9.3363\times \mathrm{t}\right)\hbox{-} \exp \left(\hbox{-} \left(\uplambda +{\mathrm{k}}_{\mathrm{b}}\right)\times \mathrm{t}\right)\right]/\left[\left(\uplambda +{\mathrm{k}}_{\mathrm{b}}\right)\hbox{-} 9.3363\right]\\ {}& +1.17\times \left[\exp \left(\hbox{-} {\mathrm{a}}_2\times \mathrm{t}\right)\hbox{-} \exp \left(\hbox{-} \left(\uplambda +{\mathrm{k}}_{\mathrm{b}}\right)\times \mathrm{t}\right)\right]/\left[\left(\uplambda +{\mathrm{k}}_{\mathrm{b}}\right)\hbox{-} {\mathrm{a}}_2\right]\\ {}& +\left[\exp \left(\hbox{-} {\mathrm{a}}_3\times \mathrm{t}\right)\hbox{-} \exp \left(\hbox{-} \left(\uplambda +{\mathrm{k}}_{\mathrm{b}}\right)\times \mathrm{t}\right)\right]/\left[\left(\uplambda +{\mathrm{k}}_{\mathrm{b}}\right)\hbox{-} {\mathrm{a}}_3\right]\Big\}\\ {}& +{\mathrm{V}}_{\mathrm{b}}\times {\mathrm{A}}_{\mathrm{p}}\left(\mathrm{t}\right)\end{array}} $$

In Eq. 3, the ^18^F physical decay constant is λ, and Ki, k_b_, and F were obtained in each patient by fitting the ^18^F-FDG tissue TAC (XLSTAT, Microsoft; Levenberg-Marquardt algorithm), ranging 7.5–57.5 min post-injection, uncorrected for ^18^F physical decay. Note that previous studies used ^18^F-FDG tissue data obtained at late dynamic PET imaging, i.e., beyond 2 h after injection, in comparison with the current ones obtained at early imaging (7.5–57.5 min post-injection) [12, 15]. However, the rationale for deriving Eq. 3 remains identical, whatever the time of acquisition (>t* after injection).

In each patient, the 5P-KMA model was implemented on PMOD software by using the whole experimental IF and tissue TAC data points acquired from injection, with very short frames including the bolus injection (version 3.0; PMOD Technologies, Switzerland) [13]. The 5P-KMA model can provide four kinetic rate constants, i.e., K_1,_ k_2-3-4_ and V_b_: K_1_ and k_2_ account for forward and reversed transport between blood and reversible compartment, and k_3_ and k_4_ account for forward and reversed transport between reversible and trapped compartment, respectively [12, 15]. The rate constants Ki and k_b_ may be computed from K_1,_ k_2-3-4_, as:

4 $$ \mathrm{Ki}={\mathrm{K}}_1\ {\mathrm{k}}_3/\left({\mathrm{k}}_2+{\mathrm{k}}_3\right) $$

5 $$ {\mathrm{k}}_{\mathrm{b}}={\mathrm{k}}_2\ {\mathrm{k}}_4/\left({\mathrm{k}}_2+{\mathrm{k}}_3\right) $$

### Statistical analysis

A normal distribution of the *α*_2_ and *α*_3_ values (Eq. 2) in the current study and in Hunter’s study could not be clearly showed for each IF time constants; therefore, comparisons between the two studies were made by means of non-parametric Mann-Whitney’s test (GraphPad Prism 6 software; two-tailed; 95% confidence level). Bland-Altman analysis was used for Ki comparison between 3P-KMA and Patlak’s analysis, as well as for further comparisons between 3P-KMA and 5P-KMA, and between 5P-KMA and Patlak’s analysis (GraphPad Prism 6 software; 95% confidence level) [18].

## Results

**Fig. 1:**
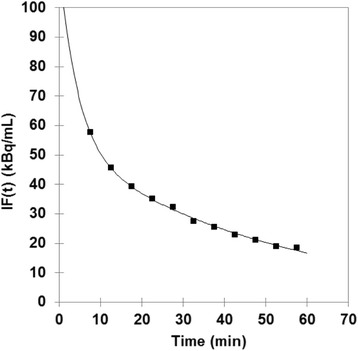
Typical IF fitting (patient 9 in Table 1; *R* = 0.989; *P* < 0.001). PET data (square) are uncorrected for ^18^F physical decay

Figure 1 shows an IF fitting in a typical patient. Values of *A*_0_, *α*_2_, and *α*_3_ are presented for each patient in Table 1. Range of IF fitting correlation coefficients over the patient series was 0.989–0.999 (mean 0.996). The *α*_2_ and *α*_3_ values found in the current study were significantly lower than those of Hunter’s study (after removing the decay correction): *P* < 0.0001 for the two comparisons.

**Table 1:** Fitting results in each patient for Patlak’s analysis, 3P-KMA and 5P-KMA (*A*_0_ in kBq mL^−1^; *α*_2_, *α*_3_, k _b_ *k*_2-4_ in min^−1^; Ki and K_1_ in mL min^−1^ mL^−1^; V_b_ no unit)

Patient	*A*_0_	*α*_2_	*α*_3_	Patlak’s Ki	3P-KMA Ki	3P-KMA k_b_	3P-KMA V_b_	5P-KMA K_1_	5P-KMA k_2_	5P-KMA k_3_	5P-KMA k_4_	5P-KMA Ki	5P-KMA k_b_	5P-KMA V_b_
1	10.82	0.1210	0.0122	0.0329	0.0312	0.0000	0.08	0.0418	0.154	0.3615	0.0000	0.0293	0.0000	0.06
2	9.16	0.2283	0.0134	0.0308	0.0312	0.0000	0.07	0.0893	0.952	0.4250	0.0000	0.0276	0.0000	0.00
3	9.57	0.1442	0.0126	0.0806	0.0845	0.0008	0.20	0.1059	0.033	0.1401	0.0000	0.0859	0.0000	0.07
4	9.69	0.1470	0.0144	0.0376	0.0391	0.0000	0.22	0.1371	0.834	0.3091	0.0000	0.0371	0.0000	0.02
5	8.28	0.1069	0.0096	0.0192	0.0198	0.0000	0.18	0.0667	0.780	0.3351	0.0000	0.0201	0.0000	0.04
6	9.55	0.1288	0.0148	0.0366	0.0390	0.0000	0.13	0.1025	0.684	0.3083	0.0000	0.0319	0.0000	0.01
7	19.79	0.2130	0.0154	0.0471	0.0560	0.0012	0.16	0.1079	0.892	0.6850	0.0000	0.0469	0.0000	0.03
8	16.26	0.1580	0.0128	0.0368	0.0384	0.0000	0.07	0.0802	1.000	0.7798	0.0000	0.0351	0.0000	0.03
9	53.45	0.2245	0.0193	0.0403	0.0446	0.0018	0.03	0.1240	1.000	0.4740	0.0000	0.0399	0.0000	0.03
10	16.46	0.1527	0.0171	0.0352	0.0360	0.0000	0.25	0.1045	0.616	0.3115	0.0000	0.0351	0.0000	0.04
11	12.45	0.1347	0.0141	0.0363	0.0358	0.0000	0.12	0.1394	1.000	0.2935	0.0000	0.0316	0.0000	0.03
12	15.14	0.1579	0.0116	0.0157	0.0202	0.0056	0.28	0.0962	0.610	0.1871	0.0099	0.0226	0.0076	0.05
13	22.17	0.1345	0.0141	0.0222	0.0249	0.0026	0.33	0.1374	0.601	0.1434	0.0043	0.0265	0.0035	0.03
14	22.41	0.1137	0.0141	0.0124	0.0135	0.0000	0.28	0.1173	0.777	0.1269	0.0071	0.0165	0.0061	0.02
15	18.61	0.2594	0.0187	0.0165	0.0171	0.0000	0.23	0.0837	0.470	0.1501	0.0044	0.0202	0.0034	0.07
16	21.40	0.1317	0.0155	0.0384	0.0389	0.0000	0.07	0.0375	0.000	0.5850	0.9596	0.0375	0.0000	0.05
17	13.53	0.1472	0.0153	0.0297	0.0266	0.0000	0.04	0.0319	0.099	0.4346	0.0000	0.0260	0.0000	0.07
18	17.28	0.1964	0.0144	0.0273	0.0323	0.0046	0.09	0.1161	1.000	0.3852	0.0031	0.0323	0.0022	0.05
19	12.26	0.1636	0.0160	0.0305	0.0314	0.0011	0.25	0.1329	0.549	0.2267	0.0052	0.0388	0.0037	0.10
20	9.37	0.1075	0.0140	0.0647	0.0643	0.0000	0.22	0.1561	0.417	0.3546	0.0000	0.0717	0.0000	0.13
21	8.26	0.1353	0.0143	0.1310	0.1450	0.0002	0.10	0.1503	0.123	1.0000	0.0000	0.1338	0.0000	0.08
Mean	16.00	0.1574	0.0145	0.0391	0.0414	0.0009	0.16	0.1028	0.5995	0.3817	0.0473	0.0403	0.0013	0.05
SD	9.82	0.0426	0.0022	0.0263	0.0288	0.0016	0.09	0.0361	0.3454	0.2247	0.2091	0.0269	0.0023	0.03

**Fig. 2:**
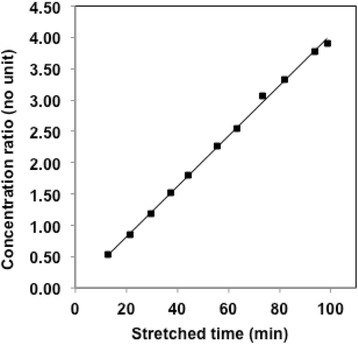
Patlak’s analysis performed in patient 9 (Table 1): *y* = 0.0403*x* + 0.0059 (*R* = 0.999; *P* < 0.001), indicating that the Ki of patient 9 is 0.0403 mL.min^-1^.mL^-1^

**Fig. 3:**
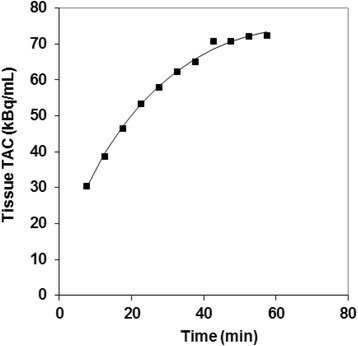
Typical 3P-KMA fitting of ^18^F-FDG tissue TAC (patient 9 in Table 1; *R* = 0.998; *P* < 0.001). PET data (square) are uncorrected for ^18^F physical decay

Figures 2 and 3 show linear Patlak’s fitting and non-linear 3P-KMA in the same patient as in Fig. 1. Values of Ki obtained from Patlak’s analysis versus Ki, k_b_, and V_b_ obtained from 3P-KMA are presented for each patient in Table 1. The range of correlation coefficients for Patlak’s and 3P-KMA fitting over the patient series was 0.971–0.999 and 0.766–0.998 (mean 0.990 and 0.958), respectively. A significant correlation was found between correlation coefficients of IF fittings and those of 3P-KMA fittings (*R* = 0.631; *P* < 0.01; graph not shown).

**Fig. 4:**
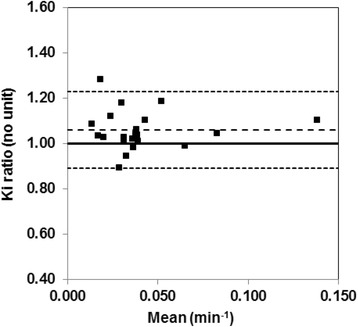
Ki ratio of 3P-KMA/Patlak against mean. Ki ratio was 1.060 ± 0.040 on average (central dashed line; 95% confidence limits not shown), with 95% limits of agreement of 0.171 (upper and lower dashed lines)

**Fig. 5:**
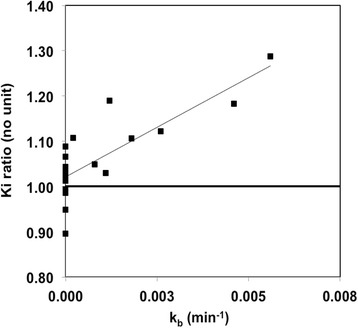
Ki ratio of 3P-KMA/Patlak versus k_b_: *y* = 43.679*x* + 1.022, *R* = 0.801; *P* < 0.001

3P-KMA Ki was found to be strongly correlated with Patlak’s Ki (*R* = 0.995; *P* < 0.001; graph not shown). Figure 4 shows the comparison between 3P-KMA Ki and Patlak’s Ki in the manner of Bland-Altman [18]. 3P-KMA Ki was significantly greater than Patlak’s analysis Ki: Ki ratio (i.e., 3P-KMA/Patlak) which was 1.060 ± 0.040 on average (95% confidence limits), with 95% limits of agreement of 0.171. When patients with k_b_ > 0 were excluded (*n* = 8; Table 1), 3P-KMA Ki was no more significantly greater than Patlak’s Ki: Ki ratio which was 1.014 ± 0.030 on average (95% confidence limits), with 95% limits of agreement of 0.098. A strong correlation was found between Ki ratio and k_b_ (Fig. 5; *R* = 0.801; *P* < 0.001).

Values of Ki, k_b_, and V_b_ obtained from 3P-KMA and values of K_1,_ k_2-3-4_, V_b_, Ki, and k_b_ (computed from Eqs. 4 and 5) obtained from 5P-KMA are presented in Table 1. Range of correlation coefficients for 5P-KMA fitting over the patient series was 0.977–0.999 (mean: 0.989). 3P-KMA Ki was found to be strongly correlated with 5P-KMA Ki (*R* = 0.989; *P* < 0.001). No significant difference was found between 3P-KMA Ki and 5P-KMA Ki: Ki ratio (i.e., 3P-KMA/5P-KMA) which was 1.017 ± 0.054 on average (95% confidence limits), with 95% limits of agreement of 0.230. No significant difference was found between 5P-KMA Ki and Patlak’s Ki: Ki ratio (Table 1) (i.e., 5P-KMA/Patlak) which was 1.056 ± 0.074 on average (95% confidence limits), with 95% limits of agreement of 0.317. 3P-KMA k_b_ was found to be significantly correlated with 5P-KMA k_b_ (*R* = 0.60; *P* < 0.01). No significant difference was found between 3P-KMA k_b_ and 5P-KMA k_b_: k_b_ difference (i.e., 3P-KMA minus5P-KMA; k_b_ ratio is not allowed since division by zero is not allowed) which was 0.00041 ± 0.00083 min^−1^ on average (95% confidence limits), with 95% limits of agreement of 0.00359 min^−1^. No significant correlation was found between 3P-KMA V_b_ and 5P-KMA V_b_ (*R* = 0.12).

**Fig. 6:**
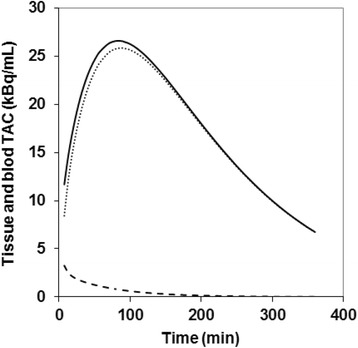
(Full) Average tissue TAC obtained from mean values of the kinetic parameters (Ki, k_b_, V_b_) found over current lung cancer series; (dotted) trapped tracer TAC; (dashed) TAC of free tracer in blood and interstitial volume. Peak time for tissue TAC and trapped tracer TAC is 84 and 88 min, respectively. Data are uncorrected for ^18^F physical decay

Figure 6 shows the tissue TAC and the part of trapped tracer and of free tracer in blood and interstitial volume (Eq. 3), by using mean values for IF and for 3P-KMA parameters (Ki, k_b_, V_b_) obtained over the current lung cancer series.

## Discussion

In each patient, 11 data points of the ^18^F-FDG IF were fitted by using a three-exponential decreasing function. These data points ranged 7.5–57.5 min post-injection, that is, after an equilibrium has been reached between compartments in order to satisfy the Patlak’s condition t > t*. This function was derived from Hunter’s results, of which *A*_0_, *α*_2_, and *α*_3_ were obtained by fitting (Eq. 2) [16]. Indeed, the relative part of each exponential function to the IF area-under-curve (i.e., the total number of molecules that are available to the tissues after injection) is 1.14, 9.62, and 89.24% (by using the mean value of *α*_1_ by Hunter and of *α*_2_ and *α*_3_ reported in Table 1), respectively. In other words, the part of the first exponential function in the whole IF, which mainly covers the IF peak, is very limited, suggesting that the mean value of *α*_1_ reported by Hunter may be used in each individual [16]. Comparison between the fitted IFs of the current study and those reported by Hunter et al. shows that the former *α*_2_ and *α*_3_ values were significantly lower than the latter ones (*P* < 0.0001) [16]. The IF fitting correlation coefficients were high (range, 0.989–0.999; *P* < 0.001; Fig. 1). The major role of a reliable analytical IF as a sum of exponential functions for implementing 3P-KMA (Eq. 3) is emphasized by the significant correlation between IF fitting correlation coefficients and those of 3P-KMA (*R* = 0.631; *P* < 0.01). It is noteworthy that, although the current study with ^18^F-FDG used a three-exponential decreasing function, 3P-KMA may also be efficient with either a mono- or a bi-exponential IF depending on the tracer. Moreover, the ways the exponentially decaying IF can be obtained may be various: either from arterial or venous blood sampling, or image-derived, or from population-based IF models possibly scaled to later dynamic measurements on blood pool ROIs [3, 19].

3P-KMA Ki was found to be 6.0% greater than Patlak’s Ki, on average, with reasonable 95% limits of agreement of 17.1%, according to Bland-Altman analysis (Fig. 4). Moreover, when patients with k_b_ > 0 were excluded, 3P-KMA Ki was no more significantly greater than Patlak’s Ki, with 95% limits of agreement of 9.8%. These findings were in agreement with Patlak and Blasberg’s comment suggesting that, in case of reversible trapping (*k*_b_ > 0), linear fitting of a concave curve rather than that of a (true) linear one (*k*_b_ = 0), results in an underestimation of the slope and hence to Ki underestimation [8]. These findings also suggest that 3P-KMA and Patlak’s analysis may be used interchangeably to assess ^18^F-FDG uptake in lung cancer lesions. However, the strong correlation between Ki ratio (i.e., 3P-KMA/Patlak) and k_b_ (and hence, between Ki underestimation by Patlak’s analysis and k_b_; Fig. 5) suggests that 3P-KMA may be more appropriate than Patlak’s analysis to accurately assess Ki in various ^18^F-FDG-positive cancer lesions, possibly showing greater trapping reversibility than lung cancer lesions [9–11].

No significant difference over the current series was found between 3P-KMA Ki and 5P-KMA Ki and between 3P-KMA k_b_ and 5P-KMA k_b_. A further comparison strengthens the current findings, between the current 3P-KMA outcomes and published ones by Dimitrakopoulou-Strauss et al., who implemented 5P-KMA in nine patients with lung tumors: Ki = 0.0414 ± 0.0288 min^−1^ (SD) and k_b_ = 0.0009 ± 0.0016 min^−1^ (SD) for 3P-KMA (Table 1) versus the mean value of 0.0304 and 0.0009 min^−1^ for 5P-KMA by Dimitrakopoulou-Strauss et al., respectively [11]. However, it should be emphasized that the measurement uncertainty of the 3P-KMA outcomes may be expected to be lower than that of the 5P-KMA one because Ki and k_b_ from 5P-KMA has to be computed by using three independent kinetic (micro)parameters (Eqs. 4 and 5). As a result, the measurement uncertainty of Ki and k_b_ from 5P-KMA combines that of the three (micro)parameters, whereas the measurement uncertainty of Ki and k_b_ from 3P-KMA may be obtained without any further combination [20]. (Note that MU of 3P-KMA outcomes was not available from XLSTAT that did not allow a possible comparison with MU of 5P-KMA outcomes.) The proposed line of argument may be associated with Galli et al.’s results showing that close values of Ki may be computed from different set of (micro)parameter values [3]. It may also explain why, unlike for the comparison between 3P-KMA Ki and Patlak’s Ki, no significant difference was found between 5P-KMA Ki and Patlak’s Ki and, hence, that the Ki underestimation by Patlak’s analysis was not revealed by the latter comparison. It may also be illustrated by the comparison of limits of agreement of 17.1 versus 31.7% that were found for the comparison between 3P-KMA and Patlak’s analysis versus the comparison between 5P-KMA and Patlak’s analysis, respectively. Furthermore, no significant correlation was found between 3P-KMA V_b_ and 5P-KMA V_b_ (*R* = 0.12), and the V_b_ mean value over the current series was found to be 0.16 ± 0.09 (SD) and 0.05 ± 0.03 (SD), respectively (Table 1). Consistently with the above-proposed comparison for Ki and k_b_, comparison of the current 3P-KMA V_b_ value of 0.16 ± 0.09 (SD) with that of 0.17 ± 0.07 (SD) previously published for 5P-KMA by Dimitrakopoulou-Strauss et al. further strengthens the findings of the current study [11].

Unlike Patlak’s analysis, the 3P-KMA approach allows expressing the whole tissue TAC as an analytical formula (Eq. 3). Therefore, at each time point, it is possible to compare it to that of its two components, that is, to the trapped tracer TAC and to the free tracer TAC. This comparison is shown in Fig. 6 by using mean values for IF and for 3P-KMA kinetic parameters that were obtained over the current lung cancer series (Table 1). Furthermore, this graph shows that the (mean) peak time for tissue TAC and trapped tracer TAC is 84 and 88 min, which could serve as landmarks to determine the optimal injection-acquisition time delay in clinical practice.

A limitation of the study is that, although the current 3P-KMA results obtained for Ki, k_b_, and V_b_ were in agreement with previous literature results by Dimitrakopoulou-Strauss et al., the k_b_/Ki ratio was low, about 2% on average in the current lung tumor series (Table 1) [11]. One could argue that 3P-KMA has been previously applied at late imaging to healthy liver that showed greater k_b_ values than those of lung tumors; however, we suggest that further studies are warranted to investigate the 3P-KMA efficiency in various tissues showing greater ^18^F-FDG trapping reversibility than lung cancer lesions [9–11, 15]. Furthermore, we also suggest that future studies should compare the performance of the 3P-KMA non-linear fitting with that of a step-wise approach replotting non-linear graphical data with different values of k_b_ in order to recover a linear fitting and hence to obtain Ki [8]. Finally, the current study did not use respiratory gating and, in the case of lesions in the lower lobes, respiratory artifacts may very likely have affected outcomes of both 3P-KMA, Patlak’s analysis, and 5P-KMA and thus might have had an influence on the reported SDs and limits of agreements.

## Conclusions

Comparison between 3P-KMA and standard Patlak’s analysis showed that the latter significantly underestimates, on average, the net influx constant (Ki) value in comparison with the former, because it arbitrarily set the release rate constant (k_b_) to zero: the greater the k_b_ value, the greater the Ki underestimation. This underestimation was not revealed when comparing 5P-KMA and Patlak’s analysis. We suggest that further studies are warranted to investigate 3P-KMA efficiency in various tissues, either physiological or pathological, showing greater ^18^F-FDG trapping reversibility than lung cancer lesions.
