# Correction to: Comparison of three-parameter kinetic model analysis to standard Patlak’s analysis in ^18^F-FDG PET imaging of lung cancer patients

**DOI:** 10.1186/s13550-019-0495-8

**Published:** 2019-03-26

**Authors:** E. Laffon, M. L. Calcagni, G. Galli, A. Giordano, A. Capotosti, R. Marthan, L. Indovina

**Affiliations:** 1 Departments of Nuclear Medicine & Lung Function Testing, CHU de Bordeaux, Bordeaux, France; 2 Centre de Recherche Cardio-Thoracique, INSERM U-1045, Université Bordeaux, Bordeaux, France; 3 Physics Unit, Fondazione Policlinico Universitario “A. Gemelli”, Roma, Italy; 4 Institute of Nuclear Medicine, Università Cattolica del Sacro Cuore, Roma, Italy; 5 Institute of Physics, Università Cattolica del Sacro Cuore, Roma, Italy; 6 Service de Médecine Nucléaire, Hôpital du Haut-Lévêque, Avenue de Magellan, 33604 Pessac, France; 7 Fondazione Policlinico Universitario A. Gemelli IRCCS, Roma, Italia; 8 Università Cattolica del Sacro Cuore, Roma, Italia

**Correction to: EJNMMI Res**

**https://doi.org/10.1186/s13550-018-0369-5**

Following publication of the original article [1], the authors flagged that the author affiliations detailed in the article are incorrect for the authors M. L. Calcagni and A. Giordano.

These authors are detailed in the article with affiliation ‘4’ (*Institute of Nuclear Medicine, Università Cattolica del Sacro Cuore, Roma, Italy*).

However, **both authors should have instead these (two) affiliations:**

*Fondazione Policlinico Universitario A. Gemelli IRCCS, Roma, Italia*

*&*

*Università Cattolica del Sacro Cuore, Roma, Italia.*
